# Pausing Mid-Sentence: An Ecological Model Approach to Language Disorder and Lived Experience of Young Male Offenders

**DOI:** 10.3390/ijerph18031225

**Published:** 2021-01-29

**Authors:** Dermot Fitzsimons, Ann Clark

**Affiliations:** Clinical Audiology, Speech and Language Centre, Queen Margaret University, Edinburgh EH21 6UU, UK; aclark@qmu.ac.uk

**Keywords:** SLCN, justice, speech and language therapy, offending, language disorder

## Abstract

International research evidence has firmly established a high prevalence of language disorder in young offender populations. Less is known about young offenders’ perspectives on their own language abilities. The study recruited an opportunity sample of 10 young men in custody at a Scottish youth offending institution who had recent experience of segregation. This mixed-methods study investigated participants’ views on their language and communication abilities to inform future support and intervention, and formal language assessment was also administered to investigate indicative prevalence of language disorder within the sample. It focused on their communication with professionals and peers in justice, education and welfare settings. Results of standardised language assessment indicated the presence of language disorder in 44% (*n* = 4) of the sample (*n* = 9). Thematic analysis of interview data led to formulation of three themes: Valuing Communication, Literacy and Learning; Exerting Control; and Seeking Support. The first theme is discussed with reference to Bronfenbrenner’s Bioecological Model. Participants offered reflective and rich views on their lived experience. They provided perspectives on features of successful interaction with peers and authority figures, importance of effective communication and the difficulties they encountered. This study argues for additional communication support for young people in the justice system.

## 1. Introduction

Young people who offend are commonly from highly disadvantaged backgrounds, live in areas with few amenities and have reduced access to educational services and employment opportunities which could otherwise further their individual development [1,2,3]. In addition, young people who offend often have complex health needs, with trauma and adverse childhood experiences such as parental abuse and neglect common in their backgrounds [4] (for a comprehensive review). These young people consequently have an increased likelihood of looked-after experience than their peers and are at greater risk of mental health conditions [5]. 

Over the past 15 to 20 years, a high prevalence of language disorder has been identified within offending populations. Cohort studies have consistently indicated that between 60 to 90% of young people who offend have a language disorder [6,7,8], compared to a prevalence of 7.5% [9] in the general population. This has received attention in research and policy literature in Anglophone countries such as the UK, Canada, USA, Australia, and New Zealand in particular. Studies have mainly focused on either establishing prevalence within groups involved with the justice system or investigating the nature and strength of the associations between language disorder, risk and background factors such as social disadvantage, social and emotional behavioural difficulties, and use of violence. Language disorders are a known risk factor for behavioural problems [10] and volatile peer relationships, with poor outcomes in particular for mental health, educational attainment and quality of employment opportunities [11,12,13,14]. Involvement in youth justice services places substantial demands on language ability as young people must use their language skills to give their ‘side of the story’, to justify decisions and interpret their own and others’ motivations [15]. 

While most young people who offend are diverted away from further criminality, a smaller but significant group become involved in more persistent and serious offending, with some going on to receive custodial sentences [16]. While in prison, some will be further involved in violence and transgression of prison rules, leading to their being removed from the main prison population and placed in a separate area of the prison with reduced social contact [17,18]. 

If the needs arising from language disorder remain unidentified and unmet, an individual’s chances of engaging effectively with criminal justice processes can be limited, thereby reducing opportunities to access support and receive fair treatment. This reduction of opportunity would amount to a contravention of UN Convention on the Rights of the Child Articles 12 (right to be heard) and 13 (right to freedom of expression) [19]. Over the last 15 years in the UK, the issue of the high prevalence of language disorder within the young offender population has gradually moved from the bounds of research literature into parliamentary and public discourse, most significantly as a result of the Bercow Report [20] into Speech and Language Therapy (SLT) provision throughout England and Wales. Specifically, Recommendation 28 (p. 11) within the report specified that the Government’s Youth Crime Action Plan should actively address the needs of this population as a matter of urgency. In Scotland, the Royal College of Speech and Language Therapists has been instrumental in placing Speech, Language and Communication Needs (SLCN) of young offenders on the political agenda [21] with a scoping study reporting that only one SLT in the whole of Scotland had ringfenced funded time with young offenders [22] in community and in prison at the time of data collection. 

Speech and language therapy has been viewed as a cost-effective service in terms of public savings that may be made by involvement of young offenders with the service [23,24]. There is some evidence that intervention with young offender groups is effective and can improve language assessment scores in the majority of cases [8,25] but studies into therapy and intervention approaches to improve young offenders’ language and communication abilities are still a rarity within the evidence base. The focus in the literature around these issues until recently has been on establishing need within the population and the nature of language and communication difficulties encountered by this population. 

It is a key notion in Speech and Language Therapy practice that access to means of effective communication is fundamental to an individual’s wellbeing; in addition, a holistic approach by professionals when considering the needs of that individual is an ethical requirement of practice [26]. In the research literature, however, while there has been much significant, valuable and necessary work on the establishment of prevalence of language disorder in the young offender population, far less is known about the personal perspectives of these young people and the extent to which they share the values of the professionals with whom they come into contact, particularly regarding their views on the value they place on their own and others’ language and communication [27,28]. These views are an essential component to the formulation of a holistic profile, and in order for intervention—whether direct or indirect—to be appropriate and effective, the perspectives of young people themselves must be taken into account. 

A suitable theoretical framework that allows a holistic view of the individual located within the relevant systems they occupy is Bronfenbrenner’s Bioecological Model. Applying this framework to an investigation of the views of young men within the criminal justice system around their interactions with others and their own abilities provides us with an opportunity to examine the experience of a group of individuals who regularly interact with a variety of institutions but whose own views and attitudes about those interactions is, to date, not well documented in the literature. 

### 1.1. Bronfenbrenner and Bioecological Systems Theory

Bronfenbrenner’s Bioecological Systems Theory (BST) emphasises the active role the individual plays in their own development while interacting within and with these systems. The theory was originally conceptualised as the Ecological Systems Theory (EST) in the late 1970s [29,30] to account for the various competing and overlapping influences on child development.

**Figure 1:**
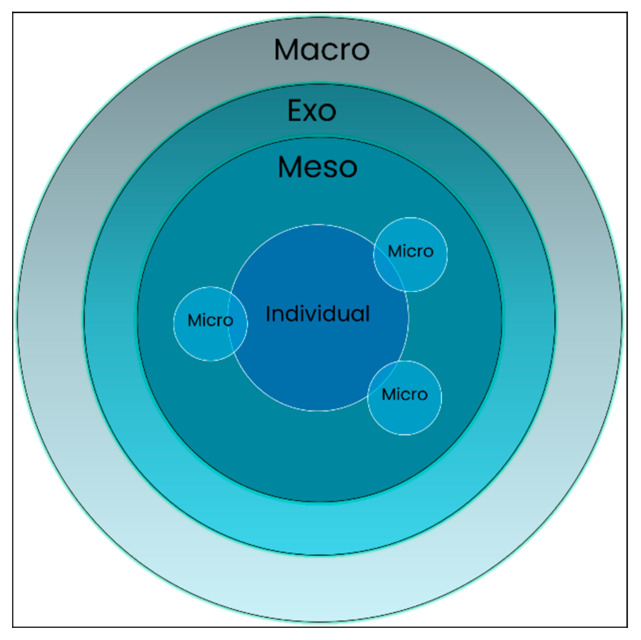
Bronfenbrenner’s “nested systems”, as conceptualised in Ecological Systems Theory [30].

Systems within the model are placed at increasingly distal levels from the individual, who is situated at the centre; this has been described by Bronfenbrenner [30] as a “set of nested structures, each inside the next like a set of Russian dolls” (p. 3) (Figure 1). 

Each of the model’s components is examined briefly below:

“Individual”: The individual is placed centrally to the model and characterised as a self-contained biological system. Genetic and biological factors influence the developmental course of the individual into adulthood. Biological, hereditary and genetic influences affect the individual’s lived experience and their development within the four hierarchical systems in which they are embedded. 

“Microsystem”: The microsystem consists of the immediate settings and environments experienced by the developing individual in which they typically have direct, face-to-face contact with other individuals. Bronfenbrenner describes microsystems as “a pattern of activities, social roles, and interpersonal relations experienced by the developing person in a given face-to-face setting...” [31] (p. 1645). Initially, a child’s microsystem will consist of the home and family/carers. As they develop, the number of institutions and individuals encountered increases and are characterised as nursery, primary school, secondary school, further education settings, and subsequently, workplaces. More specifically, courtrooms, Children’s Hearings and prisons themselves could be considered microsystems. The peer relationships encountered and nurtured in these settings are conceptualized as intrinsic to the microsystem. 

“Mesosystem”: Where multiple microsystems overlap, they exist within a mesosystem; the mesosystem is created or broadened when an individual enters a new environment. Within the welfare and justice systems, a lawyer representing their client in court, a careworker visiting a young person in prison, or family attending a Children’s Hearing may illustrate this overlap of microsystems, creating a mesosystemic interaction. The developing person is contained within both microsystems.

“Exosystem”: This further distal level is described by Bronfenbrenner as “the linkages and processes taking place between two or more settings” [32] (p. 24) where effects on the individual that emerge from the exosystem are indirect. For example, in the justice context, sentencing policy, the content of the prison education curricula, and provisions made for mental health support while in prison are all formulated within the exosystem and have indirect effects on the development and potential for development of the individual.

“Macrosystem”: The outermost layer of the Systems Model, the macrosystem describes the cultural and social norms, political systems, beliefs and values that underpin and influence the opportunities for development of the individual, in all of the other systems of the model. Rosa and Tudge [33] characterise the macrosystem’s “hallmark” as “its overarching belief system or ideology” [33] (p. 247). As an illustration of this prototypical experience, Bronfenbrenner gives the example of school classrooms, where one looks much like the other, as a result of the macrosystem “set[ting] the pattern for the structures and activities occurring at the concrete level” [29] (p. 513).

### 1.2. Bioecological Systems Theory: “Person–Process–Context–Time”

By reformulating the Ecological Systems Model into the Bioecological Systems Theory [32], Bronfenbrenner and his colleagues further described the complex, dynamic and dialectical relationship between individuals and the social environment. The four elements proposed in this model were viewed as simultaneous rather than additive or accumulative.

These components are conceptualised as:

- Process.
- Person.
- Context.
- Time.

Regarding “Process”, the main drivers for individual human development are proximal processes, defined as “enduring forms of interaction” [34] (p. 317). The authors proposed that these processes consisted of “progressively more complex reciprocal interaction between an active evolving biopsychological human organism and the persons, objects, and symbols in its immediate environment.” (ibid)

In the case of “Person”, three characteristics were described by Bronfenbrenner as influential on developmental outcomes—force, resource and demand.

Force properties are characterised as generative or disruptive. A generative force is a personal quality promoting the influence of proximal processes, for example, curiosity, responsiveness to others; initiating activity with others; ability to defer gratification in favour of longer-term aims. The disruptive characteristic is one that hinders or interrupts proximal processes, for example, “impulsiveness, explosiveness, distractibility, inability to defer gratification, or, in a more extreme form, (readily) resort to aggression and violence.” [35] (p. 1009).

Resource properties affect the individual’s ability to engage with proximal processes, with “ability, knowledge skill and experience” [36] (p. 812) promoting such engagement, while “genetic defects, low birthweight, physical handicaps, severe and persistent illness or damage to brain function” (ibid) possibly interrupting or reducing capacity to do so.

Demand characteristics are the perceived qualities of the individual by others within the individual’s social environment; these are characterised as having a knock-on effect on whether opportunities for proximal processes to influence development are offered or established. The authors [36] gave examples such as physical attractiveness/unattractiveness, hyperactivity/passivity, and type of temperament. Other personal identity markers such as perceived sexuality, age, skin colour or perceived gender may be regarded as demand characteristics that could affect access to proximal processes.

The “Context” component of the PPCT developmental framework essentially comprises the four nested systems of the earlier Ecological Systems Model (micro-, meso-, exo- and macro-). Proximal processes (the “engines of development” as described above) were conceptualised as taking place within the interpersonal microsystems level of the Context component of the model. 

The “Time” element added the developmental significance of the passage of time. The Time component was conceptualised as comprising three levels: microtime, mesotime, and macrotime, with microtime as “continuity versus discontinuity in ongoing episodes of proximal process” [35] (p. 995), i.e., the specific episodes experienced by the individual; mesotime as the frequency of these proximal process episodes over longer periods, for example weeks and months; and macrotime “focus[ing] on the changing expectations and events in the larger society, both within and across generations” [36] (p. 796). Macrotime was conceptualised as essentially the same as the chronosystem from the earlier model.

Individuals are conceptualised as having personal, genetic, biological and psychological characteristics (“Person”) and personal developmental history with its significant events such as death of a parent or starting school (“Time”). The social environment, which comprises the nested systems (“Context”) may limit or promote access to the “engines” of human development, proximal processes (“Process”). 

Application of the model provides opportunities to consider the asymmetric power relationships that characterise the interactions between the individual young person and criminal justice staff. These interactions occur in a wide array of differing criminal justice microsystems: prison, court, police station and Children’s Hearing room, to name a few; in addition, other microsystems to which the participant does not currently have access, for example, family, or community peers may also be considered and discussed. This model can provide a framework in which to examine: the individual’s views about their own language and communication abilities (“Person”) and significant past events (for example in school, offence history, previous involvement with justice institutions, looked-after experience) (“Time”), and how they view their interactions with others at the same or at differing levels of the model (for example, differences in interactions with peers in the community compared to those in prison; interactions with those who work in external criminal justice settings, or with those within the differing microsystems within the prison) (“Context”). It also aids the researcher in gaining views on the ways in which the prison as an institution and its staff attempt to alter the individual’s developmental course with positive aims by means of educational training, work parties and offering opportunities to gain academic qualifications (generative proximal processes) and to discuss factors that lead to more negative outcomes (disruptive proximal processes) (“Process”).

The microsystemic interactions in a single setting, for example the prison, may be further broken down into peer/peer interactions in cells, or in halls; peer/staff interactions in the healthcare unit, in halls or in teaching rooms or work parties. The model provides a means for participants to reflect on how they themselves have developed over time: while some interactions are recent and interconnected, occurring within the criminal justice system, police settings, court, prison, others are historical, e.g., an individual’s educational and/or looked-after experiences.

### 1.3. Research Questions

Research questions were formulated to investigate the prevalence and nature of language disorder in young people in custody with experience of removal from association, and to investigate their views on their own abilities and interactions within a wider variety of social settings than had previously been captured within the literature. 

#### 1.3.1. Research Question 1

What is the prevalence of language disorder in young people who have been recently segregated?

#### 1.3.2. Research Question 2

What do the young people think of their language, communication and literacy abilities, their interactions with peers and authority figures, historically and currently?

## 2. Materials and Methods 

The study took place at Scotland’s only Young Offender Institution, housing males aged 16 to 21 years. An opportunity sample of 10 young male offenders was recruited for the study. All participants were currently incarcerated at the YOI, and all had recent (within 2 months) experience of having been removed from association (“segregated”) in the prison’s dedicated wing. All had English as a first language, and none were receiving speech and language therapy intervention during the study. 

Participants were aged between 17;5 and 22;10 years (M = 20;1). All participants were given pseudonyms in accordance with confidentiality and anonymity requirements. Pseudonyms and ages of participants are shown in Table 1. 

All participants were of White Caucasian ethnicity. The figure is in close alignment with recent Scottish Centre for Crime and Justice Research (SCCJR) figures on the ethnicity of prisoners in Scotland where the population is 98% white [37]. Data on offence history was incomplete and not of sufficient quality to be included, however all participants had been incarcerated at least once before prior to the study.

From available data, half of the group (*n* = 5) had experienced non-mainstream educational provision. One participant had no looked-after experience (10%), and eight (80%) were reported as having looked-after experience either by self-report during interview (*n* = 3, 33%) or from Criminal Justice Social Work Reports (*n* = 3) and there was no data for one participant. Accommodation settings were reported as secure care (*n* = 3), foster care/secure (*n* = 1), foster care (*n* = 2) and residential school (*n* = 1). 

Only one participant had previous experience of receiving speech and language therapy. Incomplete data was available for current mental health and learning difficulty diagnoses; diagnoses of ADHD (*n* = 2), personality disorder (*n* = 2); “mild learning difficulty” (*n* = 1) and “anger issues” (*n* = 1) were reported.

### 2.1. Design

The study had a mixed-method design. Language assessment was carried out using the Core Language Score element of the Clinical Evaluation of Language Fundamentals battery, 4th edition [38]. Subtests were administered to gain a view as to the presence of language disorder. Subtests administered were Repeated Sentences, Formulated Sentences, Word Definitions, and Word Classes (Expressive + Receptive, to provide Total).

A 19-item justice vocabulary assessment was also administered, with participants required to explain to the best of their knowledge their understanding of key justice vocabulary terms such as “Alleged” and “Defence”. Results are reported elsewhere.

Semi-structured interview questions were constructed to align with a Bioecological Systems Theory [31] model, seeking to examine the variety and complexity of participants’ interactions and relationships. Questions encompassed a wide range of experiences at the micro- and meso-systemic levels, and, fundamentally, to gain participants’ views on the value and significance of effective communication in their lives.

The semi-structured interview schedule was a modified and broadened version of that compiled by Hopkins et al. [28], in turn was adapted from Sanger et al. [39] in their interviews with young people on court orders in the community to investigate their views on their own communication and literacy abilities. The scope of question topics for this study was broadened to encompass, in particular, prison and Children’s Hearings interactions alongside community and courtroom/police settings.

Due to the vulnerable nature of the participants, ethical approval for the study was sought from two sources: Queen Margaret University Research Ethics Committee and also the National Health Service West of Scotland Research Ethics Committee. The process in entirety took one year. Permission to carry out data collection on its estate was granted by the Scottish Prison Service. 

### 2.2. Inclusion/Exclusion Criteria

The following inclusionary criteria were applied to potential participants at the start of the recruitment procedure and for the purposes of ethical approval. Some of these criteria—while assumed by the researcher to be the case initially—are not static characteristics; the dynamic nature of attempting to meet more than once in a sometimes emotionally volatile environment, with its own social rules and behaviours meant that some participants’ suitability was continually monitored. On these occasions, advice was sought from other involved professionals before continuing with an appointment.

- At institution and accommodated within SRU within 2 months of assessment.
- Male, aged 16–21.
- English as a first language.
- Able to give consent to participate in the study.
- Able to see contents of standardised assessment materials; able to hear verbal instructions/questions as part of standardised assessment and interview.
- Willing to give their views about their communication skills.
- Assessed as presenting low risk of personal danger to those around them.

In terms of exclusions, staff compiling the list of potential participants for all recruitment rounds were made aware that potential participants were excluded from the study if English was an additional language, since the CELF-4 language assessments were to be conducted in English only.

### 2.3. Procedure

Permission was not granted by the National Health Service Research Ethics Committee to meet with prospective participants prior to their provision of consent and so initial interest in the study was provided by the young person sending back a consent form to the main prison office having read the easy-read information provided. Potential participants were initially identified by the Separation and Reintegration Unit manager who compiled a list of eligible candidates based on inclusion criteria. Prison administration staff then sent via internal mail a copy of the information sheet and consent form to potential participants with instructions to return these to the office if the young people were interested in participating. 

After this, the first author met with the participant to check understanding and answer any questions or concerns the young person might have before continuing. Participants were assured they could leave the study at any time without giving a reason at the start of each session. Semi-structured interviews were consistently conducted prior to language assessment for the study. One participant left the study post-interview prior to the administration of the CELF-4 assessment. He gave permission to use the interview data previously obtained.

All participants consented to the interviews being audio recorded. Interviews ranged in duration from 21 min to 77 min, with a mean duration of 39 min. Six hours and 27 min of interview recordings were collected. All interview audio data were subsequently transcribed by the first author and analysed using NVivo10 software (QSR International, Melbourne, Australia).

All four CELF-4 Core Language subtests were administered to nine of the 10 participants.

### 2.4. Approach to Interview Data Analysis

A thematic analysis approach [40] to the interview data was employed. The authors define thematic analysis as a “method for identifying, analysing and reporting patterns within data” (p. 79). They specify six stages to the analysis process:

Familiarising self with the data; generating initial codes; searching for themes; reviewing themes; defining and naming themes; producing the report.

Thematic analysis was appropriate, as it allowed a flexible and dynamic approach to the interview data. The first author followed this process in the course of the data analysis as closely as possible. Coding led to the formation of three superordinate themes:

- Theme A, valuing communication, literacy, and learning.
- Theme B, exerting control.
- Theme C, seeking support.

This article focuses only on Theme A as it relates specifically to long-term outcomes. Results for other themes are published elsewhere.

## 3. Results

### 3.1. Language Assessment—CELF-4 Core Language Score

Table 2 displays group scores for the Core Language Score composite language measure. The group mean (84.78) falls just within the marginal/borderline/mild impairment range (CLS = 70–85). Core Language Score index scores (50–102) indicates a scoring pattern for the group ranging from −3 SD from the mean to slightly above the expected mean performance score (102) for age equivalent 16;11 (mean = 100). 

Group Core Language Score results are summarized as follows:

- 5/9 (56%) of the group scored within normal limits (CLS 86–115).
- 2/9 (22%) of the group scored below normal limits in the Marginal range (CLS 70–85).
- 2/9 (22%) of group scored in the very low/severe range (CLS < 70).

### 3.2. Interview Data

**Figure 2:**
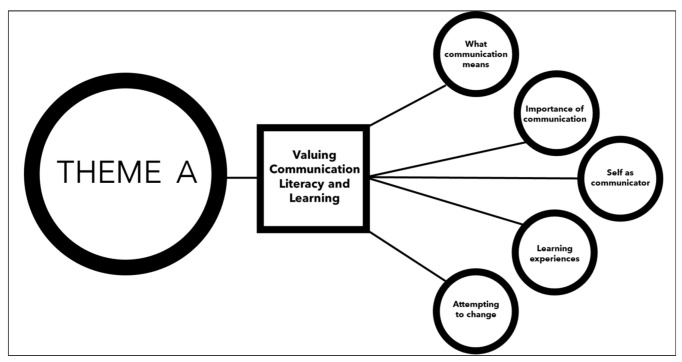
Theme A—valuing communication, literacy, and learning.

Results discussed below focus on valuing communication, literacy, and learning. Figure 2 displays its subthemes.

Participants were asked to give a definition of communication and to rate themselves as communicators. These responses are reported together as often participants would provide their reasons or further elucidation of their responses.

#### 3.2.1. Subtheme 1: What Communication Means

Participants were asked to define “communication”, then “good” and “poor” communication. A majority (*n* = 9) emphasised “speech”, “speaking” or “talking”. Half of participants included the social dimension in definitions, referring to “people”, “two people” “other people” or “others”:

Michael: “It’s how…you talk with folk and that.”

John: “How people talk to each other, interact with each other. Aye.”

*1a. “Good” communication: definitions*

When asked to define good communication in their own words some participants discussed primarily verbal means, with a sense of ease and mutual or shared understanding also featuring in their definitions:

Lucas: “Good communication—where you talk to people all the time, stuff like that.”

James: “(…) we’ll both talk and listen, and there’s a, there’s like a just a natural respect if you know what I mean?”

Michael: “Being understood and that.”

*1b. “Poor” communication: definitions*

Definitions of “poor/bad communication” were wide ranging, with some personal experiences offered. A majority of participants focused on various perceived deficits of verbal aspects of communication, with insufficient talking or information sharing:

Lucas: “Bad communication is when you just sit there, dinnae talk to anybody, tell anybody your problems, and stuff like that.”

David: “No’ talking to anybody or nothing.”

Several participants focused on pragmatic/social aspects of communication, for example, *being an arsehole* and *being cheeky* (Alan); *getting aggressive* (Mark), but also:

Stephen: “Somebody who doesnae pay attention to anybody, doesnae listen.”

Lucas: “(…) you need to talk to people, need to be able to socialise with people. You cannae just sit and keep everything to yourself. Cos that’s when things start going wrong in your head.”

While participants frequently characterised good communication in positive personal/social terms, examples of poor communication commonly described situations where speakers either did not like or were not known to each other:

James: “…if you try and communicate with someone you don’t get on with, it can be difficult.”

Stephen: “(…) if you knew the person better, that could help communication as well.”

#### 3.2.2. Subtheme 2: Importance of Communication

Participants were asked if they felt that good communication was significant or important to them. In response, participants offered a wide variety of views, some describing situations with very high personal stakes. Andrew discussed his reasons to try and be a more effective communicator:

“For me, it’s important, […] right now it is, man, cos if I just start screaming at people and that, I’m never gonna get parole, and I’ll have to dae a full six years, so for me, it’s important.”

Also, Lucas provided a highly personal account of his experience of depression and self-isolation, where he underlined his own view on the importance of communicating with loved ones:

“Good communication is important, aye. (R: How?) (…) At one point in my life, I was suicidal, and like, never telt anybody anything, and I tried to take my own life, and I woke up with tubes down my throat and everything, I was in the hospital. My mum found me foaming out my mouth lying on my bed, so I suppose aye, you do really need to talk to people sometimes.”

The remainder of responses discussed the impact at an everyday level where messages in conversation needed to be understood or everyday contact with the outside maintained:

John: “Aye, obviously it is, cos… em… (…) to get on with people and that, and obviously understand other people’s point of views, or…whatever it is you’re talking about.”

Mark: “Mm. Pretty much, if you’re not communicating well enough, like, there’s… they might not understand what you’re trying to get through to them.”

#### 3.2.3. Subtheme 3: Self as Communicator

Participants were asked to rate their communication skills on a scale of 1–5, and then prompted to expand further on their rating; some participants did not respond verbally to these prompts.

James discussed a change in his communication behaviours as a result of self-imposed pressures to be a “hardman” and working to change his thinking on this image of himself:

“Er… I’d put myself at about a 3…(…) A 3 or 4. […]it’s almost like the way I force people, they should know just how to act around me, if you know what I mean, but this is again, this is just getting in that mentality, in that hardman mentality.”

He goes on to describe the frustrations he experiences at his own—as he sees them—weaknesses in his communication skills:

“When I feel myself getting worked up, I find it hard to… verbally communicate with people, (…) I feel like I’m always having to explain the situation, and it ends up dragging on. And it never gets the response that I want, it never happens the way I want it to (…) then I get overexplaining things, and I get myself worked up, because…cos I’m overcomplicating things, they’re butting in on it, and I’m like that, no! No! No! You don’t understand what I’m saying (…)”

Alongside James, Stephen offered detailed reasons for his rating as a communicator (2½–3): he describes feelings of uncertainty and some concern that his style of communication is not effective when dealing with others:

“Between a 2½ and a 3. (R: 2½ to 3. Why do you say that?) Cos, I just feel, as if, when I’m talking to somebody, I feel as if they don’t really understand me, or… See when I say something to somebody, it’s like… I’m either speaking too fast or they cannae make out what I’m saying.”

While Martin did not offer a numerical rating of his communication skills, he offered a clear opinion about his communication strengths, and settings in which he knew he found communication more challenging:

“I’m only good at it with the people I’ve been brought up with and the pals I’ve been brought up with. Other people, nah.”

#### 3.2.4. Subtheme 4: Education Experiences

A majority of participants offered perspectives on their experiences while at primary and secondary school. The first author asked participants to describe their experience of school and whether they had experienced support.

Where provided, participants gave predominantly negative views of their interactions with school teaching staff, with what they perceived as unreasonable behaviour by teachers as the main reason. Stephen and Mark characterise interactions with teachers at school by a mutual lack of respect, and reciprocity of “attitude”, which was typical of a majority of views:

“Like, sometimes, see if teachers, pure strict teachers, I never had any luck man, they pure…pure had an attitude wi’ me, anytime I’d try and get my attitude back, man, they’d be like, “Less of that attitude…!” And I’d just be like that (sucks teeth). That’s by the time I got to high school, I started… I turned into a teenager and that, I wasnae having it.”

“[on teacher] […] he used to come in and (…) I was…just straight out of the class. Sent out. I would literally just be coming in, putting my bag on the floor again, look at him and that’d be it, it’d be straight back out. […] Just basically cos he didn’t like me.”

A majority of participants described frequent interruptions in their education as a result of removal from mainstream schooling:

Researcher: “Where did you go to school?”

David: “All different schools. Behavioural ones.”

Lucas: “Got kicked out, I was on behavioural support when I was in Primary 5, til I was…went to high school. And then in that school I was on behavioural support and missed all my days. I got kicked out for life at the start of third year. When I was 15.”

Lucas’s description of the circumstances around his removal from mainstream schooling centred on what he saw as an unjust resolution to an argument between himself and the rector of his school:

“(…) I’d go to the rector and he’d end up shouting at me, end up laughing at him and then it gets into a big heated argument and he starts spitting on me…So… I just pushed him, and got kicked out for life. Got charged with assault (laughs). He was spitting in my face… Ken like, when he shouts, spit comes out, and it’s no nice. I wouldnae spit in his face, so… Why should he do it to me?”

Two participants described their experiences of further education which had been curtailed due to involvement with the criminal justice system and subsequent imprisonment:

James: “I got myself on an access course so I changed to construction and engineering, general level, and then you get a curriculum head for the construction, and I done like 2 months of construction and engineering […] this is when I started getting in trouble with the police and going to jail, so I never finished…I finished a good like 85% of the course but I never finished it all.”

Lucas describes how he had plans to be a chef, with his course attendance was interrupted a number of times by spells in prison:

“Put me into college and got kicked out of that and then like, school wasnae for me so I went and got my NQT Qualification, I want to be a chef. And then I done half my second year, come in here, started that again, come in here, been in here a few times.”

He reported that he still had a goal of being a chef and resuming his studies on release. He spoke with enthusiasm about his favourite elements of the course and the satisfaction he got from hard work and ingenuity:

“Then we’d sit and cook like 40 covers. Starters, mains, desserts. […] It was hard going, you just keep constantly on the move. Making bread fae scraps, making all sorts. Make everything fae…next to nothing.”

#### 3.2.5. Subtheme 5: Attempting to Change

Half of participants described being motivated to change their behaviour, attempting to do so by modifying their communication style, and the language they used, and the tensions they experienced in their efforts to make these changes.

Andrew: “It’s just…cos I’m doing an LTP and I’ve got it says parole an’ that, you’ve gotta fuckin… Just the daft wee things you’ve gotta stop (…) if I just start screaming at people and that, I’m never gonna get parole, and I’ll have to dae a full six years, so for me, it’s important. … daft wee guys, they might not give a fuck in here.”

James: “…don’t even like fighting any more. It just doesn’t appeal to me.”

Stephen: “It feels if… It feels as if I belong here. I know I pretend I don’t, but it feels if…I fit in here. That’s something I’m trying to change. […] I’m gonna try… try and get out the habit of just…the routine of going back in.”

## 4. Discussion

Applying Bronfenbrenner’s Ecological Systems Theory [31] to the findings of the study allows a consideration of the complex and layered nature of the interactions participants experience in their everyday lives in the variety of settings they encounter. It allows a view of young people as social beings, as rational and conscious actors who base their decisions on their self-perceived needs, building and maintaining their social relationships according to their own value systems and attitudes.

Bronfenbrenner describes human development as a product of interaction between the environment and the individual, firstly in terms of the systems surrounding them, and in later iterations of his theory, emphasizing the processes underlining development as part of the PPCT (Process-Person-Context-Time) model [34]. This allows an understanding of human development taking place “through processes of progressively more complex reciprocal interaction” [36] (p. 797) where the young person understands the world and their role within it over time; also, an understanding of the impact and power of processes requires us to examine the relationships, time/location context and power relationships inherent in these processes.

### 4.1. PPCT: Process 

Bronfenbrenner and Morris [36] define proximal processes thus:

“Examples of enduring patterns of proximal process are found in feeding or comforting a baby, playing with a young child, child-child activities, group or solitary play, reading, learning new skills, athletic activities, problem solving, caring for others in distress, making plans, performing complex tasks, and acquiring new knowledge and know-how.”

[36] (p. 797).

Applying this concept to activities and processes reported by participants within the prison environment, we may see any activity that is regular or enduring as an example of a proximal process, for example: regularly attending and engaging with prison education or visiting projects; learning new skills in work parties such as plumbing or painting; reading books or newspapers; writing letters to family or friends; involvement in experimental research studies; playing an instrument; writing music and performing for others; talking to peers and officers about transferring to other halls or a different prison; receiving visits from family; visiting the gym with friends, or other recreational activities such as football or pool.

### 4.2. Discussion: Research Question 1

Quantitative findings for Research Question 1 are that language disorder occurred in a significant proportion of the participant group, with 44% of the group below normal limits and one participant performing above the mean for age equivalent 16;11. The presence of language disorder fundamentally reduces opportunities for young people with an already established pattern of involvement with the justice system to engage with the proximal processes inherent to the microsystems in which they interact with peers and others. As such, the young person is less likely to be engaged in those crucial formal proximal processes that intend to support their development. Generally, in the presence of other risk factors such as parental disengagement, absence of a consistent carer, or lack of key supportive authority figures, there is greater likelihood of involvement in what Johns et al. [41] refer to as “constellations of negativity” (p. 9) in the micro-and meso-systems in which they interact, i.e., involvement in more negative peer interactions, reduction in prosocial interactions with others (avoidance behaviours, confrontation behaviours).

### 4.3. Discussion: Research Question 2

Bronfenbrenner’s model can be applied in order to answer Research Question 2. Clear examples of proximal processes emerge from the interview data and subsequent themes. The school environment is a primary microsystem for proximal processes to support development. Reduced success in formal proximal processes such as education, according to Johns et al. [41] may lead to rejection of these processes by some young people in favour of—particularly in adolescence—negative peer influences, which can act to reiterate and consolidate less prosocial group identities.

Only one participant had experienced SLT involvement and his view was unclear as to why. The presence of an unsupported language disorder in children and young people is also well evidenced to increase the likelihood of disengagement from school [42,43,44], of school exclusion [45], and of reduced quality and quantity of peer interaction [46,47]; these concepts of rejection of formal proximal processes of development are borne out by the reported experiences of the young people in the study, most of whom described their schooling experiences in this theme as short, in mostly negative terms, with relationships with staff, lack of interest in the majority of subjects and removal from mainstream education cited as primary reasons.

Youth justice institutions may be seen as attempting to build positive relationships on a microsystemic and mesosystemic level (between peers, between staff and young people; between outside educational and project work and the young people) in order to counteract negative microsystemic ones (e.g., negative peer group interactions).

### 4.4. PPCT: Person

Bronfenbrenner and Morris [35] conceptualise three influential characteristics of the person that contribute to the shaping of development: disposition (individual characteristics including motivation, temperament and persistence), resources (experience, knowledge, skills and abilities), and demand (individual characteristics that invite or discourage reactions from the immediate social environment and elicit a response immediately).

“Disposition”: In the case of young male offenders, disposition may be influenced by previous experience of home, justice settings, the peer group, and so on. The young person may, due to previous experiences, have varying levels of motivation according to the setting, contributing to readiness or otherwise to engage with the proximal processes that occur within that setting, as discussed above. Many participants describe their school experiences as difficult, with frequent exclusions, leaving school before 16, or entering alternative schooling; however, a change in motivations is also observed for a number of participants where they discuss attempts to change their communicative behaviours to reduce their aggressive or destructive behaviours in the prison environment and generalise these to life outside post-liberation. Andrew, Lucas, and James all discuss their need to make changes to how they interact in the prison environment—despite reporting a different manner of interaction in community—in order to attain goals of, for example, ‘Enhanced’ status (a higher level in the Incentives and Earned Privileges Scheme), early parole, or movement to adult prison. They make use of avoidance strategies in their interactions in the prison and make decisions to engage with education and work programmes in order to prepare them better for life post-liberation.

“Resource”: Resource characteristics—experience, skills, knowledge, and abilities, but also experiences and social and material resources such as access to amenities like housing, transport, or food, and also educational or employment opportunities—may often be unseen or hidden [35,48] and “influence the capacity of the organism to engage effectively in proximal processes” [49] (p. 635). As such, low SES and poverty, affecting access to amenities and opportunities, are also conceptualised as resources in the model. An unidentified language disorder is a prominent factor in determining the degree to which individuals may engage or be able to engage with proximal processes.

Mental health conditions e.g., anxiety, depression, behavioural disorders such as ADHD or ODD all act to affect the capacity for and degree to which the individual may be able to interact substantially to allow proximal processes to influence development.

Out of the participants, 44% had an unsupported language disorder; this condition is highly likely to limit an individual’s access to the very proximal processes that could strengthen these assets and further his or her development; increased speech and language therapy provision in the justice estate would provide tailored support to provide opportunities to strengthen these assets.

“Demands”: It is well established that young people with unidentified language disorder are at greater risk of misinterpretation of their lack of understanding or reduced expressive abilities as non-compliance when dealing with authority figures and professionals in the justice system [50,51,52]. This high level of negative demand on the individual, for example, breaching bail conditions, increased violent incidents due to cultural pressures within the prison, and breaching of prison rules due to a lack of understanding, has real and long-lasting consequences for the individual.

### 4.5. PPCT: Context

In the model, the familiar concentric systems are viewed as “contexts of development” where proximal processes influence the development of the individual. Interactions within the microsystems of school and prison were the main focus of this article; not all participants discussed all interactions in all microsystems but what is clear is that a majority of participants had difficulties in historical settings, e.g., in the school environment, or were currently finding interactions within many of the microsystems they inhabited as challenging.

At the individual/microsystemic level, the importance of reflection to participants on their interactions with friends is apparent. Reflection informs consolidation and justification of self-protective, often non-proactive communication behaviours, both in community and in prison. Conversely, some participants, e.g., James, Lucas, and Andrew, described attempts to rupture this systemic influence by using an avoidance strategy both in community and prison environments, reducing contact with peer influences who might distract or manoeuvre them away from their goal of timely liberation or staying out of trouble.

### 4.6. PPCT: Time

Bronfenbrenner [49] conceptualises time as the final crucial element in individual development, emphasising the importance of the interaction being of an enduring nature: “to be effective, the interaction must occur on a fairly regular basis over extended periods of time” (p. 620).

Participant descriptions of their interactions—whether with peers, family, and professionals were often in terms of the changeability and lack of regularity of their interactions in the microsystems they inhabit: leaving and starting at different schools, the variety of care placements they encounter by type and duration, repeatedly coming into and out of prison, starting further education courses but not being able to finish them. Extended and frequent interactions within microsystems that could have direct beneficial developmental effects are rare within the interview data and subsequent themes.

Johns et al. [41] describe the importance of time and trust in changing attitudes and moving individuals towards more prosocial proximal processes of development, allowing opportunities for the young people to mature out of offending behaviour. Trust and mutual respect are considered vital pre-requisites by participants, as discussed by, for example, James in his view of good communication, and Stephen when describing his school experiences.

Most participants discussed the importance of changing their behaviours, of perceived changes in themselves in terms of their attitudes and plans while they had been in the prison, with some discussing the changes they had seen in themselves while in prison, or the desire to do so. Participants were able to describe the importance of change, of development over time, and the pressing need to do so.

The majority of participants discussed their experiences of difficulties at school, school exclusion, education in a variety of settings, looked-after experiences, emotional and behavioural difficulties, and inevitably, involvement with police and the courts before their sentencing and imprisonment. They describe repeatedly a reduced experience of successful interactions with the authority figures they encountered, from school, to family breakup and care experience, to the courtroom, to police interview, and to prison. Through the lens of Bronfenbrenner’s Ecological Systems Theory, this lack of successful interaction is mirrored in the reduced experience of positive proximal processes that may support their development throughout the micro- and meso-systemic level interactions they experience.

It is at these crucial junctures that intervention in the pathway is essential. A number of initiatives to implement intervention with SLT involvement are in existence, for example, the No Wrong Door project run by North Yorkshire County Council includes two speech and language therapists within both of its dedicated professional support teams. The team also consists of care workers, clinical psychologists and police liaison officers. The aim of the No Wrong Door initiative is to provide integrated support in the community for young people with escalating needs who are on the edge of care. In the five-month period from April to September 2016, 83 out of 142 referred young people were found to have an unidentified speech, language or communication need; the communication support workers were then able to signpost and also offer indirect intervention by offering training, consultation, and advice to other professionals and family involved with the young person. Evaluation of the initiative after two years highlighted the following outcomes: reduction in criminal activity by the young people involved, an increase in those involved in Education, Employment, or Training, and a reduction in high risk behaviours including substance use and absconding. On the basis of the success of the No Wrong Door initiative, Perth and Kinross are taking a similarly hub-based multidisciplinary approach with their REACH project which recently reported a successful multidisciplinary approach [53].

Milton Keynes Youth Offending Team has adopted a “screening out” approach to communication support needs, rather than “screening in”, where there is a base assumption that young people referred to the service will have some form of a special educational need that needs to be met in order to begin to bring about a better outcome [54]. Engagement with the services offered by the young person is a crucial aspect in the success of all of these initiatives, with the building of positive relationships between professionals and service users, and a shared understanding of common goals, a key element of any future work.

Indeed, the quantitative and qualitative findings of the study have implications for SLT practice in the prison environment. There is a still a lack of crucial speech and language therapy services in the justice and care systems, despite the now established positive effects of involvement of SLT in these areas on the lives and outcomes of the young people affected. Police [55], and Scottish Children’s Panel volunteers [56] have reported a need for further training to recognise language and communication difficulties in these young people and refer onwards.

At the moment, no specific language skills screening procedures are in place for newly sentenced and housed prisoners. A screening tool examining an individual’s receptive and expressive language skills, narrative skills, vocabulary, and literacy as they entered the prison would allow professionals to have an overview of the immediate formal needs of the individual and tailor intervention and support accordingly.

Common to all of the above examples is the screening of communication support needs at a point where the individual embarks on new interactions within microsystems that are part of exosystemic justice, prison, and care institutions. Each “system” comprises smaller, one-to-one or group microsystemic interactions that, through proximal processes, shape that young person’s future development. Whether the young person is entering or at high risk of entering care, or beginning to make use of youth offending team services, or entering prison, these are the points at which gaining detailed knowledge of their communication support needs—by use of assessment and interview data—can be most effective in informing intervention with the aim of providing better outcomes for those individuals. The need for training of staff located at all these junctures about language disorder is vital. Recent Scottish Government proposals to raise the age of referral to the Children’s Hearings system to include 16- and 17-year-olds would also provide another point of contact by which language and communication needs arising from language disorder could be recognised and met successfully.

Training on communication difficulties in vulnerable and at-risk populations can provide another means of intervening in the pathway. Need for training has been identified in the Children’s Hearings System [56] and police officers [55] with a recommendation also being made for SLT services at all points of the justice pathway [56]. An e-learning approach has been adopted by the Royal College of Speech and Language Therapists, which offers free training (The Box—www.rcsltcpd.org) for justice professionals to support them in identifying communication difficulties of the people they work with and understanding their impact on individuals involved in the justice system. Studies have shown staff training in this area has been shown to be effective [8,57,58].

Participants frequently offered insightful and reflective views about their own language and communication abilities, the importance they placed on communication and literacy, and the selectivity with which they described their motivations to communicate. Participants frequently discussed issues they felt arose around their language and communication abilities, for example, Stephen on his concerns he was not understood by his peers, or James feeling as though he was overly expressive and often took a long time to get his point across. Given the paucity of standardised assessments for this age group, and the subtlety of language difficulties in adolescence and early adulthood [59,60] using interview methods alongside language assessment when working with young people is a valid and significant addition to the information that formal assessment can provide, augments and informs a more collaborative approach between justice professionals and SLTs to goal setting if the possibility of one-to-one intervention arises. In addition, understanding the values these young men prize around mutual respect, shared understanding, having feelings of control or agency, and a need for familiarity and predictability with the authority figures and peers they encounter on a daily basis is vital to understanding their worldview and likelihood of engagement with any future language and communication interventions. Thus, the presence of language disorder in this population does not preclude these young people from offering insights and perspectives on their strengths and challenges. In accordance with UNCRC Article 12, gaining this information is vital to providing a more holistic picture of the individual and the challenges they face and should be incorporated into practice alongside traditional standardised language assessment methods.

## 5. Conclusions

The wide variety of historical and current experiences described by participants suggest that imprisoned young offenders have several crucial junction points in their lives that have ultimately led them to their current situation. This study shows the value of viewing the young person’s reports of their own lived experience through the Bioecological System lens. All participants had had contact with the justice system previously with little or no contact with Speech and Language Therapy Services. Pathways to imprisonment for young people, while individual in the sense that the circumstances that lead to imprisonment will vary for each person, are broadly similar. Key themes, features, and experiences arise between individuals which offer indications of risk. Unidentified and unmet communication support needs are a crucial component of this mosaic of risk, because of the well documented evidence surrounding the associations between unidentified needs and school exclusion and educational attainment, increased risk of mental health difficulties and increased risk of behavioural diagnoses. Identifying and meeting the communication support needs of this vulnerable group is a key element in the effort to disrupt the pathway many of these young people are travelling.

## Figures and Tables

**Table 1:** Pseudonyms and ages of participants (*n* = 10).

Pseudonym	David	Stephen	John	Michael	Martin	Andrew	Alan	Mark	James	Lucas
Age	19;8	17;5	20;11	20;9	19;11	20;11	20;6	17;10	22;10	20;3

**Table 2:** Group CELF-4 Core Language Score (mean, median, SD, range).

CELF-4 Core Language Score (*n* = 9)	
**Mean**	84.78
Median	88
SD	16.92
Minimum	50
Maximum	102
